# Convalescent plasma treatment for SARS-CoV-2 infected high-risk patients: a matched pair analysis to the LEOSS cohort

**DOI:** 10.1038/s41598-022-23200-1

**Published:** 2022-11-09

**Authors:** Noemi F. Freise, Smaranda Gliga, Johannes Fischer, Nadine Lübke, Matthias Lutterbeck, Miriam Schöler, Edwin Bölke, Hans Martin Orth, Torsten Feldt, Christoph Roemmele, Dominik Wilke, Jochen Schneider, Kai Wille, Christian Hohmann, Richard Strauss, Martin Hower, Andreas Ruf, Joerg Schubert, Nora Isberner, Melanie Stecher, Lisa Pilgram, Jörg J. Vehreschild, LEOSS Study Group, Katja de With, Christoph Spinner, Julia Lanznaster, Gernot Beutel, Norma Jung, Siri Göpel, Timm Westhoff, Bernd Hohenstein, Katja Rothfuss, Siegbert Rieg, Maria Madeleine Ruethrich, Jan Rupp, Frank Hanses, Tom Luedde, Björn Jensen

**Affiliations:** 1 Department of Gastroenterology, Hepatology and Infectious Diseases, Medical Faculty and University Hospital Duesseldorf, Heinrich Heine University, Duesseldorf, Germany; 2 Department for Transfusion Medicine, Medical Faculty and University Hospital Duesseldorf, Heinrich Heine University, Duesseldorf, Germany; 3 Institute of Virology, Medical Faculty and University Hospital Duesseldorf, Heinrich Heine University, Duesseldorf, Germany; 4 Heinrich Heine University, Duesseldorf, Germany; 5 Department of Radiotherapy and Radio Oncology, Medical Faculty and University Hospital Duesseldorf, Heinrich Heine University, Duesseldorf, Germany; 6 Department of Internal Medicine III, Gastroenterology and Infectious Diseases, University Hospital Augsburg, Augsburg, Germany; 7 University Hospital Carl Gustav Carus, Dresden, Germany; 8 Technical University of Munich, Munich, Germany; 9 Johannes Wesling Klinikum Minden, Ruhr-University Bochum, Bochum, Germany; 10 Department of Oncology and Infectious Diseases, Klinikum Bremen-Mitte, Bremen, Germany; 11 University Hospital Erlangen, Erlangen, Germany; 12 Department of Internal Medicine, Klinikum Dortmund gGmbH, Dortmund, Germany; 13 Staedtisches Klinikum Karlsruhe, Karlsruhe, Germany; 14 Elblandklinikum Riesa, Riesa, Germany; 15 Department of Internal Medicine II, Division of Infectious Diseases, University Hospital Wuerzburg, Wuerzburg, Germany; 16 Faculty of Medicine, University Clinics, Department I of Internal Medicine, University of Cologne, Cologne, Germany; 17 German Centre for Infection Research (DZIF), Partner Site Bonn-Cologne, Cologne, Germany; 18 Department of Nephrology and Medical Intensive Care, Charité - Universitätsmedizin Berlin, Berlin, Germany; 19 Center for Internal Medicine, Medical Department 2, Hematology, Oncology and Infectious Diseases, University Hospital of Frankfurt, Frankfurt, Germany; 20 Emergency Department, University Hospital Regensburg, Regensburg, Germany; 21 Hematology and Oncology Clinic II, Passau Hospital, Passau, Germany; 22 Clinic for Hematology, Hemostaseology, Oncology and Stem Cell Transplantation, Medical School Hannover, Hannover, Germany; 23 Internal Medicine Clinic I, University Hospital Tübingen, Tübingen, Germany; 24 Medical Clinic I, Marien Herne University Hospital, Ruhr-University Bochum, Bochum, Germany; 25 Nephrological Center Villingen-Schwenningen, Villingen-Schwenningen, Germany; 26 Gastroenterology, Hepatology and Endocrinology Clinic, Robert-Bosch-Hospital Stuttgart, Stuttgart, Germany; 27 Department of Infectious Diseases, University Hospital Freiburg, Freiburg, Germany; 28 Institute of Infectious Diseases and Infection Control, University Hospital Jena, Jena, Germany; 29 Department of Infectious Diseases and Microbiology, University-Hospital Schleswig- Holstein, Lübeck, Germany

**Keywords:** Diseases, Infectious diseases, Viral infection

## Abstract

Establishing the optimal treatment for COVID-19 patients remains challenging. Specifically, immunocompromised and pre-diseased patients are at high risk for severe disease course and face limited therapeutic options. Convalescent plasma (CP) has been considered as therapeutic approach, but reliable data are lacking, especially for high-risk patients. We performed a retrospective analysis of 55 hospitalized COVID-19 patients from University Hospital Duesseldorf (UKD) at high risk for disease progression, in a substantial proportion due to immunosuppression from cancer, solid organ transplantation, autoimmune disease, dialysis. A matched-pairs analysis (1:4) was performed with 220 patients from the Lean European Open Survey on SARS-CoV-2-infected Patients (LEOSS) who were treated or not treated with CP. Both cohorts had high mortality (UKD 41.8%, LEOSS 34.1%). A matched-pairs analysis showed no significant effect on mortality. CP administration before the formation of pulmonary infiltrates showed the lowest mortality in both cohorts (10%), whereas mortality in the complicated phase was 27.8%. CP administration during the critical phase revealed the highest mortality: UKD 60.9%, LEOSS 48.3%. In our cohort of COVID-19 patients with severe comorbidities CP did not significantly reduce mortality in a retrospective matched-pairs analysis. However, our data supports the concept that a reduction in mortality is achievable by early CP administration.

## Introduction

In December 2019, the novel corona virus SARS-CoV-2 (severe acute respiratory syndrome coronavirus 2) triggered a global pandemic that continues to this day, resulting in millions of deaths from severe pneumonia with acute respiratory distress syndrome (ARDS) and multi-organ failure. The widespread impact of the virus includes a severely strained health system and a global socio-economic crisis, as well as long-term health limitations following coronavirus disease 2019 (COVID-19). By 29 July 2022, more than 572 million cases of SARS-CoV-2 infection and more than 6 million deaths from the virus have been recorded^1^.

Although most patients have only mild symptoms (81%), 14% develop a severe course of COVID-19 and 5% ARDS^2^. Patients at increased risk for severe disease progression include dialysis patients, patients with cardiovascular diseases or immunosuppression, e.g., due to previous solid organ transplantation, stem cell transplantation, autoimmune disease, treatment of active malignancy or uncontrolled human immunodeficiency virus infection (HIV). Morbidity and mortality have been observed to be significantly increased in the above patients^3–5^.

At the onset of the pandemic, there were no evidence-based therapeutic options for COVID-19; to date, although several treatment options have been investigated and approved, e.g., by the U.S. Food and Drug Administration (FDA) and/or the European Medicines Agency (EMA), therapeutic options remain unsatisfactory for many patients. For example, antiviral treatment with remdesivir is not approved when estimated glomerular filtration rate (eGFR) is below 30 ml/min^6^. In light of this, the FDA published a recommendation for the use of convalescent plasma (CP) for the treatment of COVID-19 in August 2020^7^. Data on monoclonal antibodies such as bamlanivimab or the combination casirivimab/imdevimab only became available toward the end of 2020 and showed good efficacy in the early disease stage of COVID-19^8^.

Convalescent plasma has been used during pandemics caused by influenza (1918)^9^, Severe Acute Respiratory Syndrome associated Corona-Virus SARS-CoV (2003) and influenza H1N1 (2009) and is reported to have resulted in lower mortality, but the data is not very reliable^10^. Early transfusion of CP with high antibody titres is also a recommended and routine treatment for other high consequence diseases like Argentine haemorrhagic fever^11^. The underlying concept appears to be particularly promising for immunocompromised patients who have a delayed or severely reduced immune response and, after administration of CP, might be able to control the infection earlier and thus avoid disease progression^12,13^. Against this background, CP was already transfused at the beginning of the pandemic^13–16^, but always with some restraint, because of the lack of clinical data on efficacy and safety in the different phases of COVID-19 and emerging concerns that CP may promote variant selection^14,17^.

Klassen et al. performed a meta-analysis from 32 studies and 96 case series or case reports published between January 2020 to January 2021 that compared mortality of COVID-19 patients who received CP to control groups^18^. The aggregate mortality rate was lower in the transfused group than in the control groups. A major determinant was the timing of CP administration: early transfusion (within 3 days of hospital admission) with high antibody titers resulted in lower mortality^18^. Some case series report successful treatment with CP of immunocompromised SARS-CoV-2 infected patients^19–23^. Randomized controlled trials of investigational drugs usually exclude patients with severe comorbidities such as organ transplantation, active cancer, or end-stage renal disease with dialysis. However, in a pandemic, all people are affected, and data and studies on the treatment of vulnerable patient groups are especially needed.

This is a retrospective analysis of our highly selected cohort of COVID-19 patients who were hospitalized at an academic tertiary centre in Germany (UKD, University Hospital Düsseldorf) between February 2020 and February 2021 and received at least one CP. In our cohort, a significant part of our patients was immunocompromised either due to prior organ transplantation, active malignancy, or were at high risk for severe disease progression due to dialysis dependence, cardiovascular disease or advanced age. In addition, we performed a matched-pairs analysis with our cohort and two subsets of patients from the LEOSS cohort (Lean European Open Survey on SARS-CoV-2 infected patients) who did or did not receive CP.

## Results

### University Hospital Düsseldorf Cohort (UKD)

In total, 55 patients who received CP during the observation period were included in the study. The median age was 68 (range 25–100), the sex distribution was 34 men to 21 women. The total intra-hospital mortality was 41.8% (23/55) and the expected mortality according to the Coronavirus Clinical Characterisation Consortium (ISARIC-4C) Score was 26% (Range 0.3–59.1%).

UKD patient characteristics, comorbidities, received therapies as well as mortality for each subgroup are presented in Tables 1 and 2.

**Table 1:** Characteristics of LEOSS and UKD patients with CP treatment.

Variable	Nr patients		Mortality (n/% out of category)	
	LEOSS	UKD	LEOSS	UKD
Total	156	55	46	23
**Age**				
18–25 years	3	1	0	0
26–35 years	6	1	0	0
36–45 years	12	5	2 (16.7)	0
46–55 years	32	3	6 (18.8)	3 (100)
56–65 years	36	13	11 (30.1)	4 (30.7)
66–75 years	36	15	14 (38.9)	10 (66.7)
76–85 years	25	9	11 (44)	3 (33.3)
> 85 years	6	8	2 (33.3)	3 (37.5)
**Gender**				
Male	119	34	40 (33.6)	16 (47)
Female	37	21	6 (16.7)	7 (33.3)
**BMI**				
18,5–24,9 kg/m2	35	18	7 (20)	9 (50)
25–29,9 kg/m2	47	18	17 (36.2)	7 (38.9)
30–34,9 kg/m2	31	8	11 (35.5)	2 (25)
> 34,9 kg/m2	14	5	5 (35.7)	3 (60)
Unknown	29	6	6 (20.7)	2 (33.3)
**Stage at diagnosis**				
Uncomplicated phase	81	14	17 (21)	3 (21.4)
Complicated Phase	45	18	15 (33.3)	6 (33.3)
Critical illness	29	23	14 (48.3)	14 (60.8)
Unknown	1		0	
**Duration inpatient stay**				
≥ 14 days	119	36	35 (29.4)	14 (38.9)
< 14 days	36	19	10 (27.8)	9 (47.4)
Missing	1		1 (100)	
**Duration ICU stay**				
< 3	31	2	5 (16.1)	1 (50)
3–15	37	20	15 (40.5)	12 (60)
> 15 days	77	14	26 (33.8)	7 (50)
Missing	11		0	
None		19		3 (15.8)

**Table 2:** Characteristics of LEOSS and UKD patients with CP treatment.

Variable	LEOSS	UKD	LEOSS	UKD
Total	156	55	46	23
	Yes/total available values			
**Comorbidities**				
Myocardial infarction	11/153	1	5 (45.5)	0
Chronic heart failure	4/153	3	2 (50)	3 (100)
Coronary Artery disease	23/153	3	15 (65.2)	2 (66.7)
Chronic pulmonary disease	11/154	2	8 (72.7)	0
**Malignancies**				
Leukemia	9/154	0	2 (22.2)	
Lymphoma	15/153	1	4 (26.6)	0
Solid tumor	10/149	4	6 (60)	4 (100)
Previous stem cell transplantation	4/149	2	1 (25)	1 (50)
SCID	n/a	1	n/a	0
Solid organ transplantation	14/153	8	5 (35.7)	2 (25)
Heart	3	1	0	0
Kidney	11	7	5 (45.5)	2 (28.6)
**Diabetes mellitus**				
No complications	25/155	0	9 (36)	
Complications	15/153	1	10 (71.4)	0
Acute Kidney injury	20/150	5	7 (35)	1 (20)
Chronic kidney disease	36/151	44	16 (44.4)	21 (47.7)
Dialysis	5/150	13	3 (60)	7 (53.8)
New renal dialysis during CR	29/110	0	21 (72.4)	0
Autoimmune disease	n/a	8		4 (50)
Vasculitis	n/a	4		4 (100)
HIV (CD4 > 500 / µl)	n/a	1		0
Immunosuppressive therapy	34/152	17	11 (32.3)	7 (41.2)
New O2 supplementation during CO	89/153	6	25 (28)	3 (50)
Prior O2 (during UC)	1/152			
**Thrombo-embolic complications**				
(TVT, PE, abdominal thrombosis)	23/153	0	7 (30.4)	0
**Other therapies**				
Remdesivir	39/151	11	10 (25.6)	2 (18.2)
Steroids High	60/153	44	21 (35)	16 (36.4)
Tocilizumab	7/141	4	5 (71.4)	2 (50)

As previously mentioned, UKD patients were divided into the three subgroups defined by the LEOSS study group, according to the disease stage at first CP administration. Additionally, we stratified patients according to the level of respiratory support. In detail, 14 patients required no oxygen supplementation at first CP administration, 18 had low-flow oxygen requirement, 14 were receiving high-flow oxygen therapy or non-invasive ventilation (HF/NIV), 5 were intubated (IMV) and 4 received extracorporeal membrane oxygenation (ECMO) support. Patients received a median of 3 CPs (range, 1–14), with a mean of 7 days (range, 0–59) after symptom onset.

Eight patients each from the subgroups without or with low-flow oxygen therapy, six patients with non-invasive ventilation, and two ECMO patients remained stable or improved subsequent to CP administration.

In the patients in whom the disease progressed after CP administration, the disease progressed as follows: Of 6 patients without supplemental oxygen, three required low-flow oxygen and three died; of 10 patients with low-flow oxygen, five required non-invasive ventilation and five died; of eight patients with non-invasive ventilation, one was intubated and seven died; of five intubated patients, one required ECMO support and four died; the other two ECMO patients died. Accordingly, intrahospital mortality was stratified by respiratory status at the time of CP administration: 21.4% (no supplemental oxygen; 3/14), 27.8% (low-flow supplemental oxygen; 5/18), 50% (high-flow supplemental oxygen/NIV; 7/14), 80% (IMV 4/5), and 50% (ECMO 2/4). Only one documented adverse event (low fever, chills) was observed after plasma administration, and a clear association could not be established.

### Survival analysis

**Figure 1:**
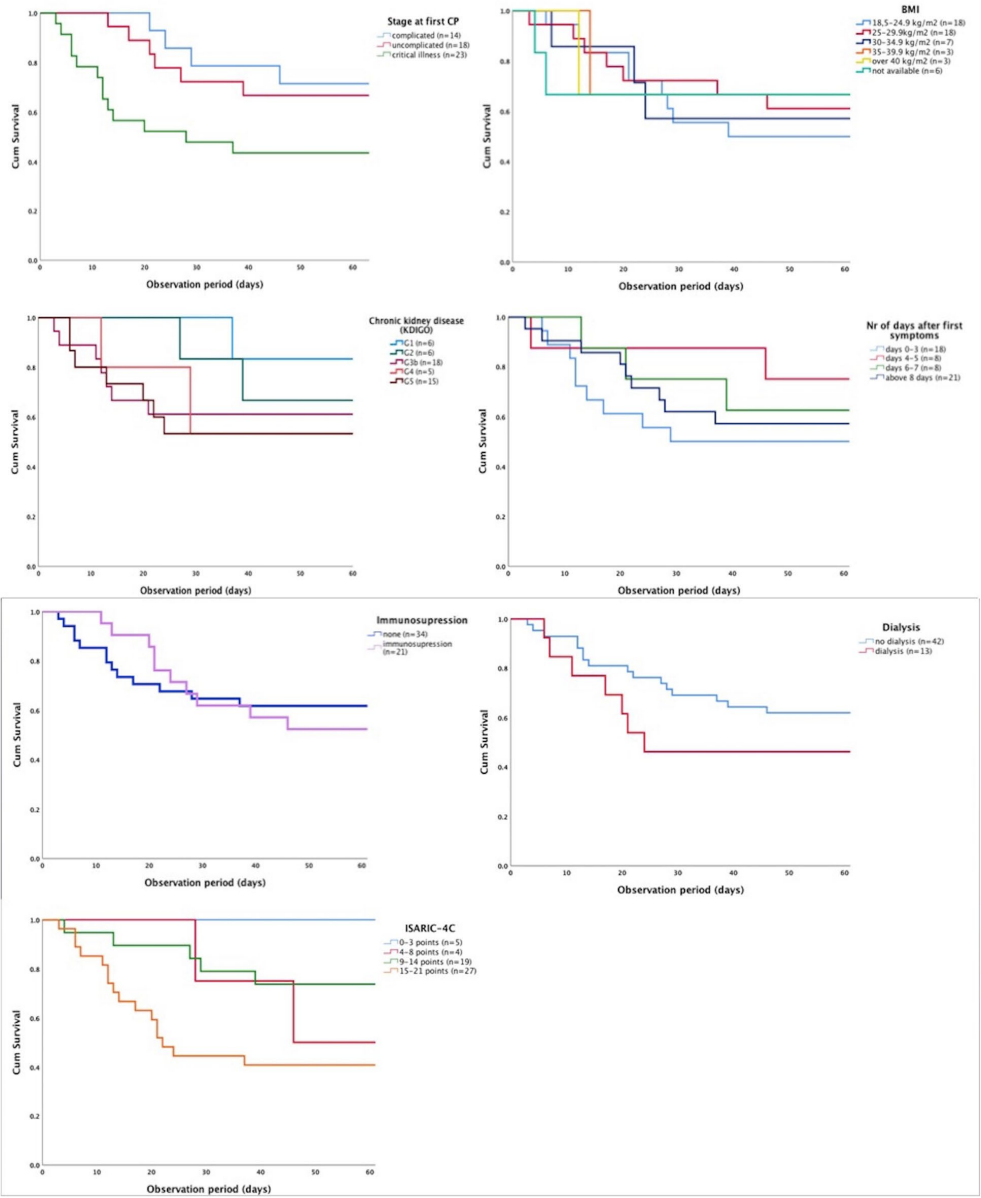
Kaplan–Meier survival plots for various parameters. *CP* convalescent plasma, *BMI* body mass index, *KDIGO* Kidney Disease Improving Global Outcomes, *ISARIC-4C* Coronavirus Clinical Characterisation Consortium.

Eighteen patients received CP therapy in the first 3 days and 21 eight or more days after onset of symptoms. Dividing the patients into four subgroups according to days after onset of symptoms at first CP administration: 0–3, 4–5, 6–7 and 8 ≥ days, we observe there is no significant difference in mortality (log-rank: 0.72) among these subgroups (The Kaplan–Meier curves are presented in Fig. 1). Out of the patients presenting in the first 3 days after onset of symptoms (*n* = 18), as documented in the patient records, only 7 were classified as uncomplicated, the rest being either in the complicated (*n* = 3) or critical phase (*n* = 8).

Patients receiving the first CP/CPs during the uncomplicated phase had higher chances of survival vs. those in the critical illness phase (log-rank = 0.007). Furthermore, patients with better kidney function and non-dialysis patients had a higher chance of survival but the difference was not statistically significant (log-rank = 0.458 and 0.224 respectively). In summary, 14/23 (60.9%) patients who received first CP/CPs in the critical phase died, compared to 6/18 (33.3%) who received CP in the complicated phase and 3/14 (21.4%) who received CP in the uncomplicated phase. The mortality in the dialysis subgroup was 53.8% (7/13) vs 38% (16/42) in the non-dialysis subgroup.

Body mass index (BMI) and the presence of immunodeficiency had no significant influence on mortality (log-rank = 0.994 and 0.767 resp.) in this cohort. Immunodeficiency was defined as the presence of following comorbidities: solid organ transplantation (heart, kidney), active malignancies, autoimmune disease, HIV, immunosuppressive therapy or corticosteroid therapy for other pathologies (COPD). Five patients had a combination of the above comorbidities. Out of the four patients with ANCA vasculitis, two patients had concurrently an active malignancy. Three patients with vasculitis had received B-cell depleting agents or chemotherapy in the months before SARS-CoV-2 diagnosis. The remaining patient with vasculitis was also a kidney transplant patient and had an untreated carcinoma.

None of the patients with an ISARIC-4C score (*n* = 5) under 3 died vs. 16/27 patients with a very high score (15–21 points) (log rank = 0.23). The ISARIC-4C score for patients receiving first CPs during the uncomplicated phase ranged from 0.3 to 40.1%. Out of the five patients with ISARIC-4C score under 3, only two patients did not have other risk factors such as reduced kidney function, immunodeficiency or advanced age.

There were only ten patients who did not have lung infiltrates at administration of first CP/CPs. The mortality rate in this small subgroup was 10% (1/10).

### Evolution of SARS-CoV-2 antibodies after transfusion of convalescent plasma (UKD)

With the exception of two patients who received three CPs the same day, no more than two CPs were administered at once. There were 23 patients with a single CP/CPs administration out of which six died (mortality: 26%) and 32 patients who received multiple CP/CPs administration. The mortality in the latter subgroup was 53% (17/32) (p = 0.054; OR 0.69; CI 95% 0.47–1.02). In total patients received a median of three CPs (range 1–14).

There was a statistically significant increase in titre after the first CP/CPs administration but afterwards there is little variation in antibody titre (Fig. S1a). There is a significant effect of time on antibody titre, especially between baseline and values two, three and five. In regard to the final outcome, patients who died had a lower antibody titre at value two and four time points (Fig. S1b). The four patients with ANCA vasculitis, who had previously received chemotherapy with B-cell depleting agents or were currently on immunosuppressive therapy did not achieve a significant antibody titre (< 5) after the first CP administration, and this titre rapidly decreased, or no antibodies were detected in follow-up measurements. Patients who presented 8 or more days after onset of symptoms had higher antibody levels at baseline that those presenting in the first 7 days (Fig. S1c).

### LEOSS cohort: patients with CP therapy

**Figure 2:**
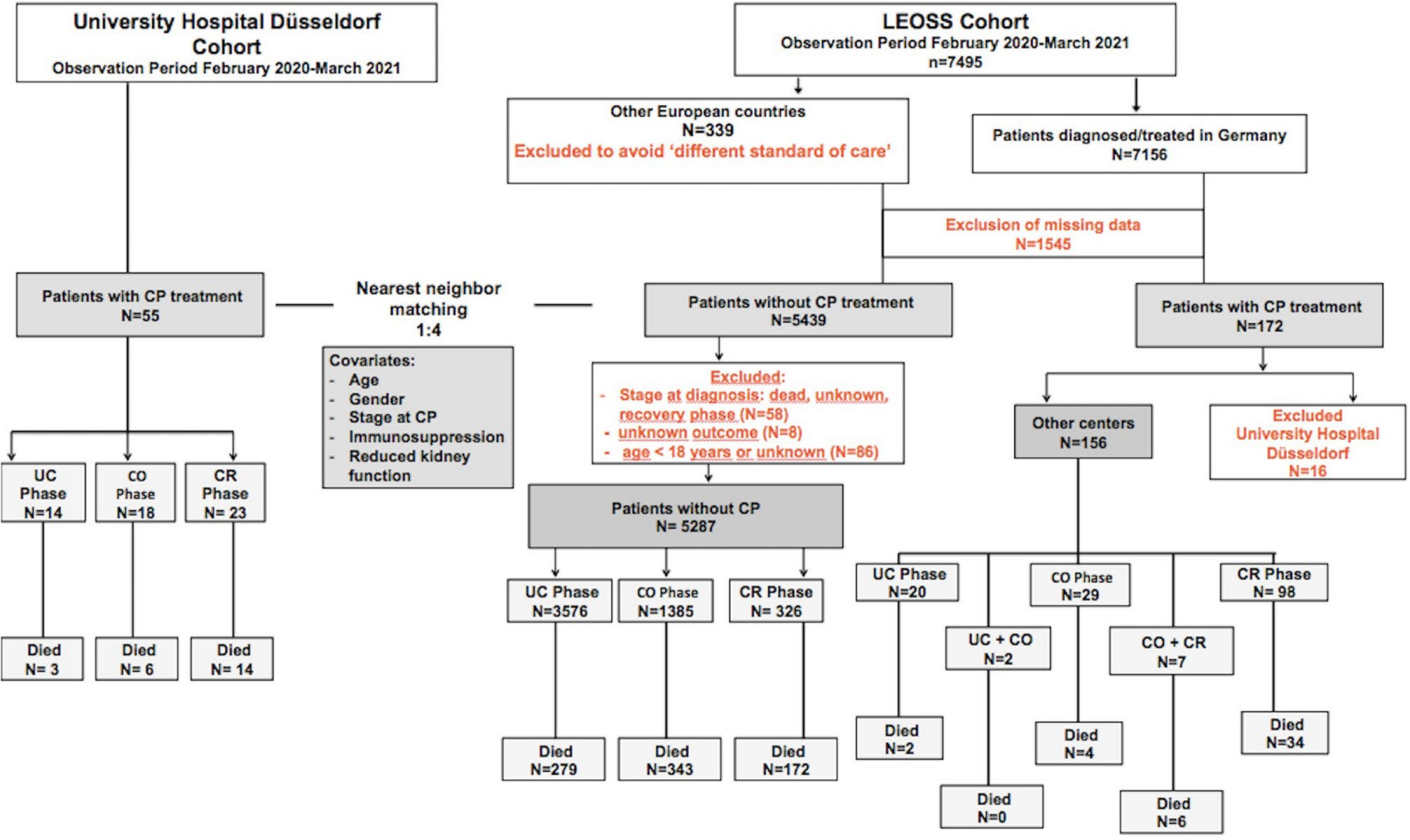
Study design and study population. *N* number, *CP* convalescent plasma, *UC* uncomplicated, *CO* complicated, *CR* critical.

Out of the 156 patients who received CP therapy, 98 patients were in the critical phase. The COVID-19 associated mortality rate for these patients was 34.7%. Two patients received CPs both in the uncomplicated and complicated phase, none died. Seven patients received CPs in the complicated and critical phase, out of which 6 died (Fig. 2). For patients who received CPs in the uncomplicated phase the mortality rate was 10% (2/20); in the complicated phase: 27.8% (10/36). The total mortality rate for patients who received CP therapy was 29.5%.

Patient’s characteristics, comorbidities and therapies are presented in Tables 1 and 2.

### Factors associated with mortality, length of hospital and ICU stay for UKD and LEOSS cohort

In the UKD cohort the factors introduced in the regression model were stage at CP, reduced kidney function, immunodeficiency and age (R^2^ = 0.294). Dialysis was analysed separately to avoid multicollinearity (R^2^ = 0.018) (Table 3). In the binary logistic regression analysis, the factors associated with mortality in the UKD cohort were the disease stage at first CP administration and immunodeficiency.

**Table 3:** Binary logistic regression to identify risk factors for death.

Demographics	UKD Cohort			LEOSS CP cohort	
	OR	95% CI for OR		OR	95% CI for OR
Stage at first CP	6.2**	1.87–20.5		1.92	0.96- 3.82
Reduced kidney function	1.7	0.98–3.07		0.98	0.41–2.39
Age	1.5	0.77–3.4		1.7**	1.22–2.32
Dialysis	1.8	0.54–6.65		12.09***	4.42–33.07
Immunosuppression	8.16*	1.35–49		0.63	0.27–1.49

In a multinomial logistic regression analysis (data not shown in Tables 3, 4, 5), patients receiving the first CP therapy during the uncomplicated phase had a 3.6 higher chance of survival in the UKD cohort (95% Confidence Intervall (CI) for Odds Ratio (OR) 1.02–13.14, *p* = 0.046). Since only a small number of patients was included in subgroups (age, kidney function, BMI), there was no significant association between a specific subgroup and mortality.

**Table 4:** Binary and multinomial logistic (stage at CP) regression to identify risk factors for longer hospital stay (**≥ **14 days).

Demographics	UKD Cohort		LEOSS CP Cohort	
	OR	95% CI forOR	OR	95% CI forOR
	Longer hospital stay			
**Stage at first CP**				
UC	1.65	0.42–6.46	1.1	0.47–2.59
CO	5.00*	1.44–17.24	1.6	0.80–3.07
CR	1.09	0.48–2.47	7.9***	4.22–14.8
Reduced kidney function	0.75	0.48–1.19	2.39	0.89–6.43
Immunosuppression	2.28	0.57–8.14	0.94	0.37–2.38
Age	1.01	0.77- 1.78	1.09	0.83–1.44

**Table 5:** Multinomial logistic regression to identify risk factors for longer ICU stay (3–15 and > 15 days).

Demographics	UKD Cohort		LEOSS CP Cohort	
	OR	95% CI for OR	OR	95% CI for OR
**ICU stay 3–15 days**				
Immunosuppression	1.46	0.39–5.51	1.69	0.47–6.02
Age	0.98	0.95–1.03	0.96	0.65–1.44
Dialysis	1.6	0.37–6.92	1.13	0.33–3.89
**ICU stay > 15 days**				
Immunosuppression	1.52	0.35–6.63	1.27	0.33–4.96
Age	0.98	0.94–1.02	0.76	0.49–1.18
Dialysis	1.02	0.19–5.53	1.58	0.41–6.17

*p < 0.05, **p < 0.01, ***p < 0.001; *UKD* University Hospital Düsseldorf, *OR* odds ratio, *CI* confidence interval, *UC* uncomplicated, *CO* complicated, *CR* critical phase.

According to the strength of association, the factors introduced in the regression model for the LEOSS cohort were the stage at first CP administration, dialysis, immunodeficiency and age (*R*^2^ = 0.220). Reduced kidney function was analysed separately. Among LEOSS patients receiving CP treatment, factors associated with death were dialysis and age and to a lesser extent stage at first CP. In a multinomial logistic regression analysis, patients receiving the first CP therapy during the uncomplicated phase had an 8.4 higher chance of survival in the LEOSS cohort (95% CI for OR 1.96–36.8, *p* = 0.04). The OR and confidence intervals CI for ORs are presented in the Tables 3, 4, 5.

Most patients, both in the UKD and LEOSS cohort had a long hospital stay (**≥ **14 days): 36/55 and 119/156 respectively and median/long ICU stay (3–15 days or above 15 days).

### Matched-pairs analysis of UKD CP cohort vs. LEOSS cohort without CP

The propensity score matching was performed using five variables: age, gender, stage at CP, immunosuppression and presence of reduced renal function. Four controls were matched to one case. There was no difference in survival between the UKD and LEOSS matched cases. Patient in the UKD cohort had longer hospital and ICU stays (Table 6).

**Table 6:** Outcome comparison in the UKD-LEOSS matched pairs.

Total nr	UKD (cases)	LEOSS (controls)	*p*-value
	55	220	
Survival	Died/Total (%)	Died/Total (%)	0.345
	23/55 (41.8)	75/220 (34.1)	
Hospital stay			**0.031**
< 4 days	2 (3.6)	31 (14.1)	
4–13 days	18 (32.7)	86 (39.1)	
≥ 14 days	35 (63.7)	103 (46.8)	
ICU stay			** < 0.001**
None	19 (34.5)	46 (20.1)	
< 3 days	2 (3.6)	82 (37.3)	
3–15 days	20 (36.4)	42 (19.1)	
≥ 15 days	14 (25.5)	38 (17.3)	

*For 11 patients in the LEOSS controls cohort, data regarding ICU stay was not available. They were not included in analysis.

Significant values are in [bold].

## Discussion

Treating COVID-19 patients with complex and severe comorbidities, such as previous organ transplantation, immunosuppressive diseases or medications is still a challenge.

This retrospective analysis presents a highly selected cohort of 55 patients with diverse but severe and complex underlying diseases who required treatment at a tertiary academic centre. Often, these patients with severe comorbidities are excluded from trials, especially prospective or randomized trials, e.g. randomized trial from Sekine et al.^24^.

21 of 55 patients were immunocompromised due to previous solid organ transplantation, active malignancy, autoimmune disease or HIV infection. The remaining patients had a variety of other risk factors, most of which also affect immune function and influence disease progression and mortality. One patient was a young woman with a SCID syndrome (severe combined immunodeficiency), the case is also separately presented by Keitel et al.^19^. Another patient was infected by HIV-1 that does not necessarily lead to a severe disease course; this depends on the treatment status and concomitant diseases, but he suffered also from histiocytosis X^25,26^.

Common comorbidities present in the patients treated with CP from the LEOSS cohort were immunodeficiency (solid cancer, solid organ transplantation, hematologic cancer) and reduced renal function. Performing a matched-pairs analysis was a challenging task because the 55 patients in the UKD cohort had complex pre-existing conditions that were difficult to account for in matching. This is one limitation of our study.

Our retrospective analysis showed an in-hospital mortality of 41.8% in our UKD cohort and a mortality of 29.5% in the LEOSS cohort of patients who received CP treatment. In the matched-pairs analysis there was no significant difference between the UKD cohort and matched controls from the LEOSS cohort without CP treatment. The mortality in our cohort is higher than the in-hospital mortality of COVID-19 patients in Germany of 22% reported by Karagiannidis et al. in September 2020^27^. The literature search revealed an increased mortality rate in immunocompromised patients with COVID-19. Martinez-Urbistando et al. showed that illnesses causing immunodeficiency are an independent risk factor for increased mortality^5^. Mehta et al. studied mortality rates in COVID-19 patients with active cancer and found a higher overall mortality rate of 28%; 25% in solid cancers and 37% in haematological cancer^28^. A meta-analysis from Belsky et al., reviewed the disease course of immunodeficient patients infected by SARS-CoV-2 and found that solid organ transplant patients, who suffer from multiple comorbidities, are more likely to require intensive care, which is a surrogate for mortality^4^. E.g., in our cohort 7 patients with previous kidney transplantation had a mortality rate of 28.6%, in accordance to our results, Alberici et al., Yilmaz et al. and Akalin et al. found mortality rates of 28%, 23% and 25% in kidney transplant recipients with COVID‐19^29–31^. However, the mortality rate in our cohort was lower than in LEOSS patients with prior renal transplantation and CP therapy (45.5%).

On the other hand, a compromised immune response to the pathogen due to immunosuppression may also protect against the organ destroying cytokine storm caused by SARS-COV-2 and lead to a favourable outcome^32,33^. But the study of Minotti et al. included mostly children or adults with rare immunodeficiencies in contrast to our cohort^33^. Regarding comorbidities and age, observational cohorts from Yilmaz et al., Alberici et al., Mehta et al. and Belsky et al. are much more comparable to our cohort^4,28,30,31^. But they reported smaller case series and all patients had the same underlying disease. Nevertheless, the overall mortality rate was still higher in our cohort, assuming due to the complex comorbidities.

In our study, patients receiving repeated CP transfusions had higher mortality rates, which might be explained by the fact that repeated CPs were given more frequently to severely ill patients who did not improve clinically and were less likely to be given to stable or improved patients.

A meta-analysis by Klassen et al.^18^ showed positive effects regarding mortality and morbidity when transfusion of CP is performed in early disease stage. Mortality was 10% in patients in our cohort who received plasma without radiologically detectable pulmonary infiltrates, presumably because CP administration occurred early in the disease. In the LEOSS cohort, mortality was similar at 10% when CP was transfused in the uncomplicated phase. CP was administered in the UKD cohort on average 7 days after onset of symptoms, only 18 out of 55 patients received it within 3 days after onset of symptoms but there was no significant difference in mortality rate. A diagnosis in the critical phase of disease and thus late CP therapy resulted in a mortality rate of 60.8% in our cohort. This can be explained partly by the high risk of death due to severe comorbidities in this selected group of patients. However, in the late phase of COVID-19, the SARS-CoV-2 virus plays only a minor pathophysiological role compared to the hyperactivation of the immune system, so that a primarily antiviral intended therapy such as CP could also be expected to be of little benefit.

Another influencing factor is the antibody titre of the CP. Joyner et al. were able to demonstrate that mortality and morbidity are only reduced if the CP have an antibody titre of at least 1:2560 (SARS-CoV-2 spike subunit 1 protein)^18,34^. Joyner et al. could also show that a lower risk of death after transfusions with high antibody titres was present only in the group of patients who were not mechanically ventilated^34^. Other studies support these findings: In CP recipients treated within 72 h of symptom onset who had high antibody titres (SARS-CoV-2 S IgG titres > 1:3200), the relative risk reduction was 73.3%, compared with recipients with antibody titres of less than 1:3200, for whom the risk reduction was only 31.4%^35^. Similar results were obtained by Salazar et al., who found a significant reduction in 28-day mortality when CP was administered within 72 h of hospital admission and the anti-IgG to spike protein titre was ≥ 1:1350^36^. Sekine et al. found no significant difference in 28-day improvement after CP transfusion, but transfusion was performed late in the disease stage when 0_2_ supplementation was already required and at least 10 days after symptom onset, confirmed by detection of neutralizing antibodies^24^.

Regarding the use of CP in immunocompromised patients with severe COVID-19, Gupta et al. reported 10 kidney transplanted patients with severe COVID-19 according to the WHO Interim Guidance^37^, 9 of whom recovered completely^38^. Transfusion of CP in our high-risk cohort at an early stage of disease resulted in a low mortality rate of 10% and, in a larger cohort, may also have shown a significant benefit in reducing mortality.

Matched-pairs analysis showed a significant longer stay in ICU and in-hospital compared to the LEOSS cohort that has not received CP. The selection of patients in the UKD cohort can in part explain the significant longer stay in ICU and in hospital caused by a higher awareness to this patient group by expecting complications, including those that may not occur directly as a result of COVID-19. In addition, patients with haematological diseases needed more time for viral clearance as also described by Avanzato et al. and Mira et al.^12,13^.

However, it must also be considered that CP administration could have a negative impact on overall survival. Systematic reviews and meta-analyses of a total of more than 10,000 patients showed no significant negative effect in terms of mortality, use of mechanical ventilation, clinical improvement or clinical deterioration^39^.The observation in our cohort that no serious adverse events, e.g., allergic reactions, were observed is supported by other publications that consider transfusion of CP to be a safe and a low-risk procedure^40^.

The ISARIC-4C score is also a good predictor of mortality in this cohort of high-risk patients. Accordingly, 46 of 55 patients were classified as high-risk (more than 9 points). All patients with an ISARIC-4C score under 3 survived and 16 of 27 patients with a very high score (15–21points) died. It is noteworthy that most of our patients had a high ISARIC-4C score, although this did not take into account the immunodeficiency and impaired renal function that were present in a large proportion of our patients. Also, young patients with severe comorbidities or rare life-limiting diseases usually achieve only a low score, so that their individual risk is not adequately reflected by the score^19,41^. In our view, these factors explain the higher in-hospital mortality in our cohort compared with that predicted by the ISARIC-4C score; in contrast, the association between a high and very high score and the outcome is clearly evident in our cohort (OR 4.07, 95% CI 1.14–14.6).

There are few reports of virus development in severely immunosuppressed patients, e.g., with deficiency of both B and T cells^14,17^. On the one hand, especially in the absence of specific monoclonal antibodies until the beginning of 2021, the indication for CP therapy was obvious in these patients due to their impaired own immune response and their risk profile. However, it should be taken into account that especially in the context of immunodeficiency and high-replicative viral variants, viral evolution after therapy with CP and monoclonal antibodies, in particular bamlanivimab, has been reported^17,41,42^.

The analysis is limited because of several factors. Since the study was performed at an academic tertiary centre, many patients were referred from secondary and tertiary hospitals, so some patient data on symptom onset and timing of SARS-CoV-2 infection were missing. Defining of onset of symptoms has not been exactly defined at the beginning of the pandemic. Therefore, in some cases, the timing of symptom onset was apparently assessed in the emergency department based on shortness of breath or clinical worsening, rather than with regard to early, mild symptoms such as sore throat, headache, or fever. Furthermore, antibody titres of the CPs were not routinely tested, so positive effects by high antibody titres in CP may not be documented. Because of the lack of a prospective study protocol, our results have many other limitations since we cannot account for multiple possible confounders or aspects such as immortal time bias. Further, another factor to mention is the rather small size of the cohorts, so the results should be interpreted within this context. In addition, the diversity of comorbidities in the UKD cohort makes it difficult to extrapolate results to the general population.

Larger cohorts would be needed to better assess the impact of CP therapy.

We hypothesize that any potential beneficial impact of CP therapy on morbidity and mortality in this highly selected group of patients is masked by the fact that in most cases CP was administered at an advanced stage of the disease.

Available data suggest that CP administration, especially when given early, holds promise for more rapid clearance of the virus and amelioration of the severity of the disease course. Considering that CP administration is not associated with high risk based on available data, CP administration should be considered as an early available and promising option for future viral pandemics or viral variants until specific therapies become available. This is especially true for vulnerable patient populations at high risk for severe disease progression or with contraindications to alternative treatment options, as represented by the patient population in our cohort.

## Methods

### Patient cohort

Retrospective cohort study of 55 patients with diagnosed SARS-CoV-2 infection (defined as detection of SARS-CoV-2 RNA from naso-pharyngeal secretions) admitted to the infectious diseases unit or intensive care unit (ICU) at the UKD between February 2020 and 25th February 2021, who received CP from cured SARS-CoV-2 donors within the framework of an individual treatment option after informed consent. None of the patients had received a vaccination against SARS-CoV-2.

The main objectives were to describe the pattern of CP use and to analyse in-hospital mortality, length of hospital stay, and ICU stay. Second, we aimed to describe the evolution of SARS-CoV-2 antibody titres after CP administration.

### Matched pairs analysis to LEOSS cohort

The Lean European Open Survey on SARS-CoV-2 infected patients (LEOSS) is an ongoing multicentric study collecting epidemiological and clinical information on SARS-CoV-2 disease.

We aimed to compare demographical parameters and clinical outcomes between our cohort and LEOSS patients receiving CP.

Furthermore, in order to evaluate if CP influences our primary outcomes, we performed a matched-pairs analysis using as cases our cohort of CP patients and as controls, LEOSS patients without CP therapy.

For this purpose, LEOSS patients were divided into two groups: patients without CP therapy and patients with CP therapy. To avoid statistical bias, LEOSS patients diagnosed and treated in countries other than Germany and patients with missing data were excluded, as described in Fig. 2.

Patients were matched based on age, sex and confounding factors influencing the outcome (disease stage at first administration of CP, reduced renal function and immunosuppression).

### SARS-CoV-2 stages of disease

Patients in our cohort were divided into three subgroups according to the disease stage at first administration of CP. The disease stage definitions were retrieved from the LEOSS cohort: uncomplicated, complicated and critical phase. The characteristics of each phase have been defined in previous publications^43^.

### Confirmation of SARS-CoV-2 infection

#### Isolation of viral genomic material and SARS-CoV-2 quantification

Respiratory samples from nasopharyngeal swabs were used for total nucleic acid extraction using the EZ1 Virus Mini Kit v2.0 on an EZ1 Advanced XL (Qiagen, Germany) according to manufacturers’ instructions. SARS-CoV-2 was detected as previously described by Corman and colleagues^44^ with a plasmid-standard for quantification^45^, by the cobas® SARS-CoV-2 test on the cobas®6800 system (c6800, Roche Diagnostics), or by the SARS-CoV-2 test on the NeuMoDx™ platform (NDX, Qiagen).

#### Detection of SARS-CoV-2 antibodies

SARS-CoV-2 antibodies were detected using the Elecsys® anti-SARS-CoV-2 (Cobas, Roche Diagnostics, Mannheim, Germany), which uses a protein representing the nucleocapsid (N) antigen in a double-antigen sandwich assay format. The assay was performed according to the manufacturer’s instructions and has a sensitivity of 85.3% 7–13 days after confirmed diagnosis (positive PCR) and 99.5 at more the 14 days after diagnosis^46^. Testing of SARS-CoV-2 anti-Spike (S) antibodies was only available after introduction of the SARS-CoV2 vaccines in early 2021. The testing of neutralizing antibodies was not routinely available during the study period.

SARS-CoV-2 antibodies were measured at baseline, either hospital admission or before administration of CP and after administration of CP. For patients receiving multiple plasmas, repeated measures were available. After CP administration, the available values were grouped in intervals of days after baseline to facilitate statistical analysis: 0 to 5 days, 6 to 10 days, 11 to 15 days, 16 to 20 days and 21 to 25 days after baseline.

### Convalescent plasma

#### Collecting plasma

CP was collected from patients with confirmed SARS-CoV-2 infection. All plasmas were collected in the Institute for Transfusion Medicine, University Hospital Düsseldorf according to the following criteria. The convalescent plasma donors donated 650 ml, 750 ml or 850 ml of plasma. Plasmapheresis was performed according to Standard Operation Procedure of the Institute for Transfusion Medicine. One plasma unit had a volume of 190 ml to 350 ml, depending on body weight of the donor. The production of convalescent plasma was subject to the same criteria as plasma products for transfusion purposes (approval according to Arneimittelgesetz (AMG), paragraph 13). As described in the permission of the blood donation services high sensitive PCR for hepatitis virus A, B, C, E, West nile virus according to the recommendation of the Paul Ehrlich Institute and HIV was performed. The donors for CP had to meet the following criteria in accordance with the guidance of the European Union on collection of COVID-19 CP: Informed written consent to donate plasma; previous infection with SARS-CoV-2 as documented by either a positive PCR (from nasal or nasopharyngeal swab, bronchoalveolar lavage or stool or a past medical history suggestive of COVID-19 and presence of anti-SARS-CoV-2 antibodies with a documented date of first positive test; proven clearance of SARS-CoV-2 from nasopharyngeal mucosa by one negative PCR result from nasal swabs or nasopharyngeal swabs and an interval of at least 4 weeks since resolution of SARS-CoV-2 associated symptoms or an interval of at least 4 weeks since resolution of symptoms of SARS-CoV-2 infection. Time and types of symptoms were documented as well as time of resolution, as well as most severe clinical status according to 9 point ordinal WHO scale; no residual severe organ dysfunction due to COVID-19; negative test for antibodies against HLA class I, class II and HNA-antigens in female donors with a history of pregnancy; Anti-SARS-CoV-2 antibodies detectable in a neutralization assay (NT) titer of > 1:160 (measured at Institute for Virology, University Duesseldorf).

#### Transfusion of convalescent plasma

Patients received 1–2/3 units of AB0-matched CP, dependent on bodyweight and depending on the volume status. CP was transfused over 30 to 60 min. During transfusion, patients were monitored, at least by continuous measurement of oxygen saturation. All patients or their legal representatives gave written informed consent to the transfusion of CP.

### Statistical analysis

Data were analysed using SPSS Statistics for Mac OS version 25.0 (IBM Corp., Armonk, USA). Simple frequencies, description and survival analysis were performed. A non-parametric Mann–Whitney test was used to assess differences in antibody titre after plasma administration. A two-way analysis of variance (ANOVA) was used to test associations between time, primary outcome and antibody titre. Bivariate correlations were used to determine associations between different parameters. Binary and multinomial logistic regression models were used to determine predictors of major outcomes. Odds ratios and 95% CI for odds ratio were calculated to assess the strength of association and statistical significance. Significant outcome predictors in both cases and controls groups were introduced as covariates in a case–control matching model. The case–control matching was performed using propensity score (nearest-neighbour 1:4) matching with R statistic^47,48^. Differences in outcome between matched cases and controls were assessed using an unpaired t-test. Differences were considered statistically significant at two-tailed *p* < 0.05.

### Variable definition

For the logistic regression and variance analyses some variables had to be regrouped. In the UKD cohort, immunosuppression was defined as the presence of following comorbidities: solid organ transplantation (heart, kidney), active malignancies, autoimmune disease, HIV, immunosuppressive therapy or cortisone therapy for other pathologies (for example COPD). Acute kidney injury (AKI) and chronic kidney disease (CKD) were regrouped as a single variable entitled reduced kidney function.

In the LEOSS cohort receiving CP the final outcome was regrouped as binary (dead/alive); malignancies/organ transplantations/stem cell transplantation/ other immunosuppressive therapies were grouped together as Immunosuppression, much like in the UKD cohort. Prior and new dialysis were grouped as a single variable named dialysis.

### Declaration of accordance to relevant guidelines and regulation

We can confirm that all methods were carried out in accordance with the relevant guidelines, treatment recommendations, and regulations available at the time of use of convalescent plasma in the cohorts presented.

### Ethical considerations

The ethical committee of the Heinrich-Heine-University Duesseldorf approved the retrospective analysis of the cohort (Study number: 2021-1385). The investigator board of Lean European Open Survey on SARS-CoV-2 infected patients (LEOSS) approved the matched-pair-analysis. Approval for LEOSS was obtained by the applicable local ethics committees of all participating centers and registered at the German Clinical Trials Register (DRKS, No. S00021145).

## Supplementary Information

Supplementary Legends.

Supplementary Figure 1.

## Data Availability

The datasets generated and analysed are available from the corresponding author on reasonable request. Datas of the LEOSS cohort are available from the LEOSS study group.

## Abbreviations

SARS: Severe acute respiratory syndrome
ARDS: Acute respiratory distress syndrome
HIV: Human immunodeficiency virus
FDA: U.S. food and drug administration
eGFR: Estimated glomerular filtration rate
CP: Convalescent plasma
UKD: University hospital duesseldorf
LEOSS: Lean european open survey on SARS-CoV-2 infected patients
ISARIC-4C: Coronavirus clinical characterisation consortium
