# Supplementary material for: Convalescent plasma treatment for SARS-CoV-2 infected high-risk patients: a matched pair analysis to the LEOSS cohort
Source: Sci Rep. 2022 Nov 9;12:19035. doi: 10.1038/s41598-022-23200-1 (PMC9643921; doi:10.1038/s41598-022-23200-1)
Supplement: Supplementary file 1 — Supplementary Legends. [file 41598_2022_23200_MOESM1_ESM.docx]

**Figure S1.** a) Evolution of SARS-CoV-2 antibodies during the observation period. b) Two-way Anova for mixed models with Tukey comparison of antibody values alive vs died subgroups. c) comparison of antibody values at baseline according to days after onset of symptoms. * p<0.05; **p<0.01, **** p<0.0001, ns: not significant, baseline: admission value or value right before CP administration.
