# Supplementary figures and images for: Convalescent plasma treatment for SARS-CoV-2 infected high-risk patients: a matched pair analysis to the LEOSS cohort
Source: Sci Rep. 2022 Nov 9;12:19035. doi: 10.1038/s41598-022-23200-1 (PMC9643921; doi:10.1038/s41598-022-23200-1)

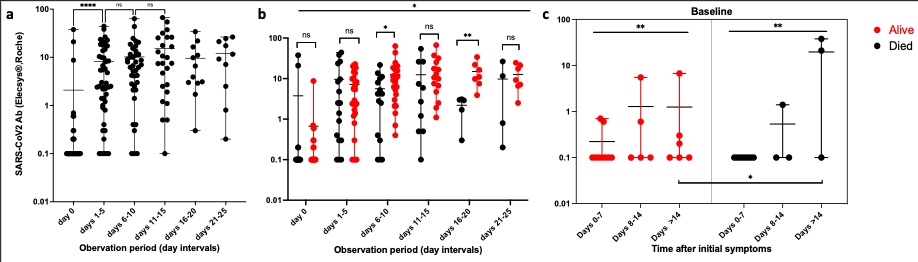

Supplement: Supplementary file 2 — Supplementary Figure 1. [file 41598_2022_23200_MOESM2_ESM.jpg]
